# Retraction: Rapamycin Inhibits Proliferation of Hemangioma Endothelial Cells by Reducing HIF-1-Dependent Expression of VEGF

**DOI:** 10.1371/journal.pone.0234141

**Published:** 2020-07-21

**Authors:** The PLOS ONE Editors

Following publication of this article [1], concerns were raised about Fig 5 and Supporting Information S1 Fig. Specifically:

- The p70S6K panel in Fig 5A in [1] appears similar to Fig 2C, VE-cadherin panel, lanes 1–4, in [2]. The corresponding author stated that an error was made in preparing Fig 2C of [2] and that Fig 5A of [1] reports the correct data.
- β-actin panel lanes 4–6 in S1 Fig of [1] appear similar to Fig 1, FHs74Int IκB panel, lanes 1–3 in [3], with aspect ratio adjusted. The corresponding author stated that these panels show different images, and further commented that the result in S1 Fig was confirmed in Fig 1A of [1].
- Harvard Medical School, Harvard School of Dental Medicine, and Beth Israel Deaconess Medical Center reviewed concerns about certain data reported in this article, confirmed the above issues, and noted that the corresponding author did not identify reliable primary data to support the results.

Harvard Medical School, Harvard School of Dental Medicine, and Beth Israel Deaconess Medical Center recommended retraction of the *PLOS ONE* article.

The corresponding author provided electronic images of blots and laboratory records relevant to the panels in question, but the files provided did not resolve the above issues and were not sufficient to support the results of the entire experiment represented in each figure. The corresponding author said he cannot provide the original raw data supporting Fig 5A, S1 Fig, and other results reported in this article because he no longer has access to it. He said that the raw data files were stored on laboratory computers at Beth Israel Deaconess Medical Center when he (DM) left the institution in 2012.

In light of the above concerns and in line with the institutions’ recommendation, the *PLOS ONE* Editors retract this article.

BRO did not agree with retraction. DM did not agree with retraction, noted that he stands by all findings and conclusions reported in this article, and said that he has initiated further actions in response to this case.

The p70S6K panel of Fig 5A is similar to material from [2], published in 2011 by Biochemical Society, which is not offered under a CC-BY license. The β-actin panel in S1 Fig includes content similar to material in [3], published in 2008 by The Rockefeller University Press and made available under a CC BY-NC-SA 3.0 license six months after publication. The p70S6K panel of Fig 5A and the β-actin panel of S1 Fig are therefore excluded from this article’s [1] license. At the time of retraction, the article [1] was republished to note these exclusions in the figure legends and the article’s copyright statement.
